# C-753-04. Multicenter Analysis of Standard of Care versus Oxandrolone versus Testosterone in Burn Hypermetabolism (MASCOTS)

**DOI:** 10.1093/jbcr/irag033.025

**Published:** 2026-04-06

**Authors:** Aaron Hong, Parker M Hannan, Anastasiya Ivanko, Denise M Danos, Alexandra D Davis, Gina L DeFelice, Jonathan E Schoen, Jeffrey E Carter, M Victoria P Miles

**Affiliations:** University Medical Center Burn Center, Louisiana State University Health Sciences Center, New Orleans, LA; Louisiana State University Health Sciences Center School of Medicine, New Orleans, LA; University Medical Center Burn Center, Louisiana State University Health Sciences Center, New Orleans, LA; Louisiana State University Health Sciences Center, New Orleans, LA; University Medical Center New Orleans, Louisiana; University Medical Center Burn Center, Louisiana State University Health Sciences Center, New Orleans, LA; UCI Health Regional Burn Center, New Orleans, LA; University Medical Center Burn Center, Louisiana State University Health Sciences Center, New Orleans, LA; University Medical Center Burn Center, Louisiana State University Health Sciences Center, Department of Surgery, New Orleans, LA

## Abstract

**Introduction:**

After a major burn injury, patients experience a hypermetabolic response leading to extensive immunosuppression, inflammation, and muscle wasting. Historically, oxandrolone has been used to combat these deleterious effects in severely burned patients. However, in June 2023, the FDA abruptly removed Oxandrolone from the market. Other anabolic supplements have shown some promise in inducing a similar blunting effect of the hypermetabolic response; however, no research has directly compared these anabolic alternatives to oxandrolone. This study aims to compare outcomes in patients receiving oxandrolone, testosterone, or the standard of care (SOC, no anabolic supplements).

**Methods:**

An IRB-approved, multicenter retrospective and prospective study is currently reviewing patients >18 years of age who suffered >20% total body surface area (TBSA) thermal burn injuries from 6/2022 to 9/2025. Patients taking hormonal modulating therapy and non-thermal injuries were excluded. Primary outcome was inpatient weight loss >5 kg. Propensity score-matched comparison was conducted between patients treated with oxandrolone or testosterone and SOC. Inpatient weight loss in the matched cohort was compared using logistic regression. Analysis was conducted in 9/2025 by the primary site.

**Results:**

The study included 527 patients: 199 received oxandrolone, 59 received testosterone, and 269 received SOC. Full thickness burns were present in 90.3%, with 19.2% having an inhalation injury and 12.5% experiencing concomitant trauma. The median hospital length of stay was 36 days for oxandrolone, 36 days for testosterone, and 25 days for SOC patients (*p*<.0001). Median absolute weight change from admission to discharge was -4 kg in oxandrolone, -2.4 kg in testosterone, and -0.25 kg in SOC patients, with a significant difference between the three groups (*p*=.0002). 110 patients treated with oxandrolone or testosterone were propensity score-matched to SOC controls; the odds of >5 kg inpatient weight loss was not significantly different (*p*=.1126).

**Conclusions:**

MASCOTS is the first study in the literature comparing outcomes of various anabolic steroids in the treatment of burn hypermetabolism. This analysis gives an overview about how anabolic supplements could affect outcomes in severely burned patients. As this study progresses and our dataset grows, we hope to further explore the effects of these anabolic agents on the hypermetabolic response and overall burn patient outcomes.

**Applicability of Research to Practice:**

This project showcases the effect of anabolic steroids in the treatment of burn hypermetabolism. The results of this study can be used to guide treatment in burn critical care.

**Funding for the study:**

Spirit of Charity Burn Foundation.

